# Machine learning-based diagnosis and risk factor analysis of cardiocerebrovascular disease based on KNHANES

**DOI:** 10.1038/s41598-022-06333-1

**Published:** 2022-02-10

**Authors:** Taeseob Oh, Dongkyun Kim, Siryeol Lee, Changwon Won, Sunyoung Kim, Ji-soo Yang, Junghwa Yu, Byungsung Kim, Joohyun Lee

**Affiliations:** 1 Department of Family Medicine, Kyung Hee University Hospital, Seoul, Republic of Korea; 2 Department of Electrical and Electronic Engineering, Hanyang University, Ansan, Korea; 3 School of Electrical Engineering, Hanyang University, Ansan, Korea

**Keywords:** Risk factors, Cardiovascular diseases, Epidemiology

## Abstract

The prevalence of cardiocerebrovascular disease (CVD) is continuously increasing, and it is the leading cause of human death. Since it is difficult for physicians to screen thousands of people, high-accuracy and interpretable methods need to be presented. We developed four machine learning-based CVD classifiers (i.e., multi-layer perceptron, support vector machine, random forest, and light gradient boosting) based on the Korea National Health and Nutrition Examination Survey. We resampled and rebalanced KNHANES data using complex sampling weights such that the rebalanced dataset mimics a uniformly sampled dataset from overall population. For clear risk factor analysis, we removed multicollinearity and CVD-irrelevant variables using VIF-based filtering and the Boruta algorithm. We applied synthetic minority oversampling technique and random undersampling before ML training. We demonstrated that the proposed classifiers achieved excellent performance with AUCs over 0.853. Using Shapley value-based risk factor analysis, we identified that the most significant risk factors of CVD were age, sex, and the prevalence of hypertension. Additionally, we identified that age, hypertension, and BMI were positively correlated with CVD prevalence, while sex (female), alcohol consumption and, monthly income were negative. The results showed that the feature selection and the class balancing technique effectively improve the interpretability of models.

## Introduction

Cardiocerebrovascular disease (CVD) was the leading cause of death in the United States in 2016, accounting for more than 900,000 deaths^1^. CVD was also the number one cause of death in Korea, accounting for 52,616 deaths in 2019^2^. The most prominent causes of death are vascular in nature, and stroke is currently the second leading cause of death worldwide^3^. Stroke burden rapidly increased for individuals up to the age of about 80 years and is the dominant cause of neurological burden for individuals between the ages of 60 and 84 years, more so in men than women^4^. The World Health Organization (WHO) has underlined the importance of lifestyle, such as tobacco use, unhealthy diet habits, physical inactivity, and psychological stress in the explosion of cardiovascular disease in the Western world, and the WHO states that three quarters of all CVD mortality may be avoided by adequate coordinated prevention actions^5^. The risk of developing CVD is reduced through early detection and lifestyle intervention. For individual patient treatment, physicians are ready to identify who is at risk for CVD. However, the challenges faced by physicians become clear when trying to screen thousands of potentially high-risk patients. Analytical techniques are needed to support mass CVD screening.

Machine learning (ML) approaches have been applied to predict various diseases and analyze risk factors based on large population datasets. Logistic regression (LR) is one of the most widely used multivariate linear models for medical data analysis. For example, LR has been used to analyze the association between family history and diabetes based on the Korea National Health and Nutrition Examination Survey (KNHANES)^6^. LR has also been used to analyze the association between muscle strength and factors of cardiovascular disease^7^. However, there are significant limitations on LR analysis. The first is that LR cannot solve nonlinear problems. Therefore, it requires an assumption of linearity between input variables and outcome. Additionally, LR requires no multicollinearity between variables. The multicollinearity indicates that some variables are highly correlated. Multicollinearity increases the standard error of odds ratio analysis, leading to incorrect relationship analysis between variables and outcomes^8^. However, these assumptions are rarely satisfied with real data. As an alternative to LR, nonlinear ML (e.g., support vector machine, random forest) has been used for data analysis. Nonlinear ML can identify complex patterns among multiple variables without any assumptions. One study obtained high classification performance in diabetes classification using ML and rural Chinese data^9^. Other studies developed highly accurate ML-based CVD classifiers using UK biobank data or the National Health and Nutrition Examination Survey^10,11^.

The development of ML has resulted in higher-performance models than pre-existing CVD risk models^12^. However, the ML models are refer to as “black-box” so it is hard to explain how the algorithm derived a specific outcome. Feature importance-based explanation has been used to describe how the ML models depend on particular risk factors. Recent studies identified that major risk factors for CVD were age, systolic blood pressure, and BMI by using the permutation feature importance (PFI) and mean decrease impurity (MDI) of tree ensemble models^13,14^. Other studies analyzed risk factors for CVD using Shapley additive explanation (SHAP) and PFI on the Korean national health insurance service screening database^15,16^. However, most ML-based studies overlook the effect of multicollinearity and irrelevant variables on risk factor analysis. Although multicollinearity does not affect the predictive power of ML models, variables with high collinearity offset the importance of each other, consequently leading to an erroneous evaluation of the importance of the variables. As a solution for multicollinearity, principal component analysis (PCA) transformation has been used in medical data analysis^17,18^. However, it is not an appropriate way to conduct risk factor analysis with PCA because it breaks up the informativity of original variables. A methodology that eliminates multicollinearity and does not decrease the interpretability is required for appropriate risk factor analysis. As much as multicollinearity, irrelevant variables are also detrimental to ML-based data analysis. They slow down the processes and lower the generalization performance and interpretability of ML^19^. For the relevant variable selection, MDI-based selection has been used with the XGBoost model^11^. Compared to these studies, we propose effective pipelines to reduce the effects of multicollinearity and irrelevant variables on risk factor analysis.

This study aims to develop ML-based CVD classifiers: multi-layer perceptron (MLP), support vector machine (SVM), random forest (RF), and light-gradient boosting machine (L-GBM) without multicollinearity and redundant variables. We used two-stage variable selection, variance inflation factor (VIF)-based filtering, and the Boruta algorithm to remove multicollinearity and redundant variables. VIF-based filtering has the benefit of eliminating multicollinearity while maintaining interpretability of the model. Further, the Boruta is an empirical and reliable algorithm that utilizes a statistical significance test to determine outcome-relevant variables. In addition to feature selection, we applied class balancing strategies to increase the confidence score of the classification models. For the model interpretation, we identified key risk factors of CVD using Shapley additive explanations (SHAP), which is a post hoc model interpretation technique that is theoretically based on the Shapley value^20^. We identified the key risk factors of CVD and the direction of the relationship between the risk factors and CVD using SHAP. In addition, we ablated feature selection (i.e., VIF and Boruta) and data balancing techniques to show how they affect to SHAP analysis.

## Methods

### Study design

KNHANES (2007–2018) is a legal survey on the health behavior, the prevalence of chronic diseases, and nutritional status of Koreans. The KNHANES is a complex, stratified, and multi-stage probability cluster survey in which the participants were not randomly selected from the Korean population^21^. KNHANES also includes sample design errors, inequality sampling ratios for interest factors, nonparticipation errors, and non-response errors. Therefore, it is necessary to consider complex sampling weights to reduce the biases between the estimators and population parameters. KNHANES provides complex sampling weights of each participant to allow data researchers to correct for these biases. Previous studies have analyzed CVD risk with logistic regression using the complex sampling weights^22,23^. In this study, we present a method for applying complex sampling weights to ML model-based data analysis.

**Figure 1:**
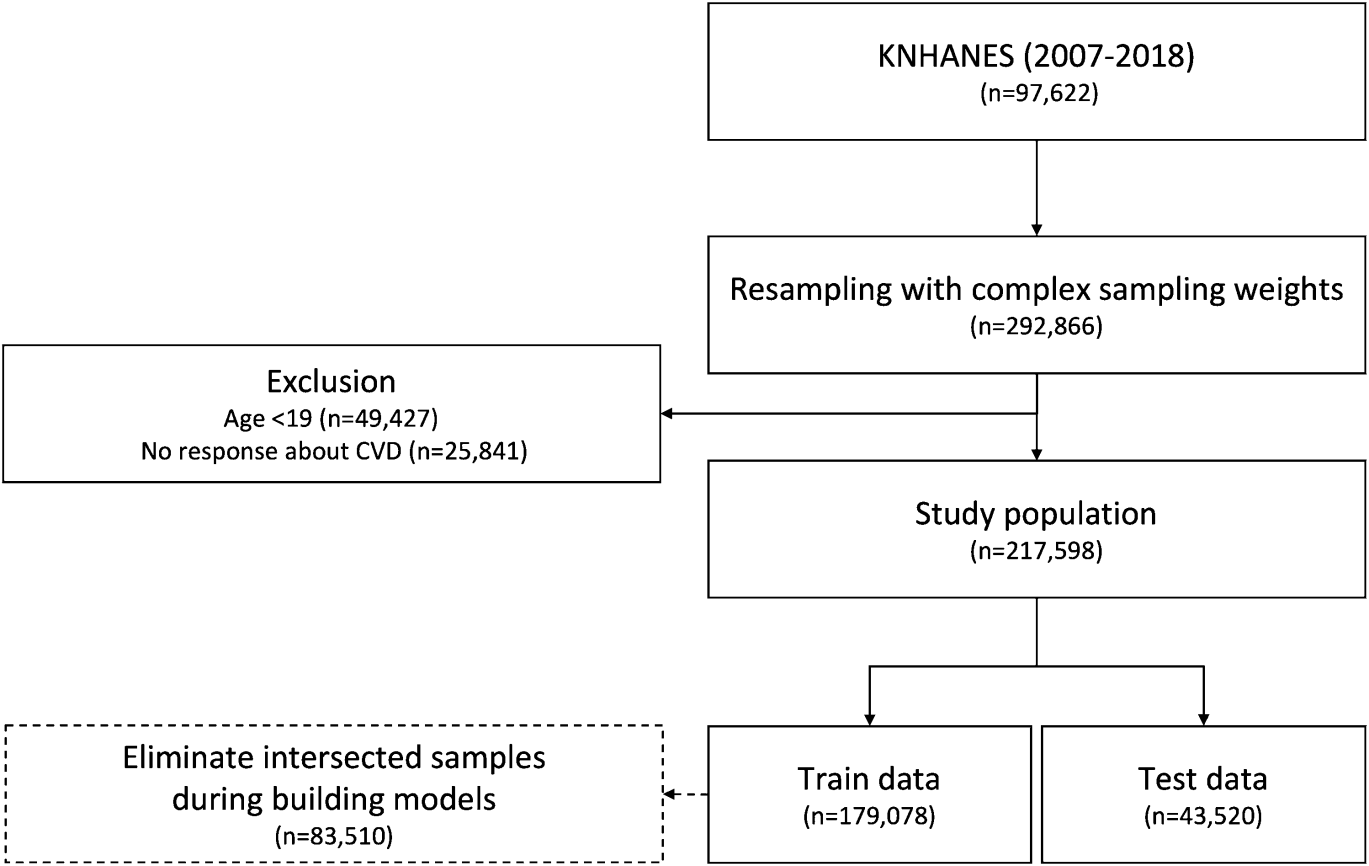
Flow diagram of the number of study participants.

We collected 97,622 samples with health questionnaire, health examination, and *itvex* variables (a complex sampling weight for health questionnaire and examination variables) from KNHANES 2007-2018. The *itvex* corresponds to the number of people that an individual represents in population, and the sum of *itvex* equals the South Korean population size (49,631,475 people). Following the KNHANES data analysis guideline, we integrated data from 2007 to 2018 and divided *itvex* by 11.5. Then, we duplicated the dataset proportional to each individual’s *itvex*. Since 49.6 million people are too many to be analyzed by ML models, we limited the total number of duplicated samples by three times (n $=$ 292,866) to the original dataset. Then, those who were <19 years (n $=$ 49,427) and those with no response about CVD (n $=$ 25,841) were excluded. As a result, 217,598 participants were selected as the study population. The participants were randomly divided into a training set (n $=$ 174,078 (80%)) and a test set (n $=$ 43,520 (20%)). As a result of duplication, some samples were selected in both the training and test sets. We removed them before the feature selection and model training (n $=$ 83,510). The flow diagram of data preparation is shown in Fig. 1. We conducted this study using Python 3.8 (https://www.python.org/) and its compatible open-source packages.

### Primary outcome

The primary outcome of this study was determining the occurrence of CVD in each participant. CVD was defined based on participants’ responses to the checklist survey questionnaire, where they were asked to indicate whether they were ‘Diagnosed by a doctor and currently suffering from the disease’ for myocardial infarction, angina pectoris, or stroke on the KNHANES Health Survey. Participants with at least one myocardial infarction, angina, and/or stroke were defined as the CVD class, and participants without any of the three diseases were defined as the non-CVD class.

### Data preprocessing

From the KNAHANES 2007-2018, we extracted the health questionnaire and examination variables that are collected in every year. Among them, we selected 34 variables that are related to CVD^24^. We refined variables to match the input format of the ML model. Binary responses were transformed to 0: a negative and 1: a positive variables. Numerical variables were redefined so that their quantitative values were consistent with their qualitative meaning, e.g., Stress perception 0: Little, 1: A little, 2: Much, and 3: Very much. Then, Z-normalization was applied to numerical variables so that the deviation of the variables did not affect classification and model interpretation. Z-normalization is processed by:

1 $$\begin{aligned} z = \frac{x - \mu }{\sigma } \end{aligned}$$

where *x* is the raw variable, $\mu$ is the mean of the variable *x*, and $\sigma$ is the standard deviation of the variable *x*. Like previous medical data studies, we replaced the missing values with the mode for binary data and the median for the numerical data^25^.

### Variable selection

Before we develop ML-based CVD classifiers, we removed multicollinearity and CVD-irrelated variables. Multicollinearity indicates how much information is shared among the variables and it makes difficult to distinguish how much each variable influenced the regression or classification^26^. We detected the multicollinearity by calculating VIF:

2 $$\begin{aligned}&\text {Total\,sum\,of\,squares\,(TSS)} = \sum _{i=1}^{N}(Y_{i}-\bar{Y_{i}})^{2} \end{aligned}$$

3 $$\begin{aligned}&\text {Error\,sum\,of\,squares\,(ESS)} = \sum _{i=1}^{N}(Y_{i}-\hat{Y_{i}})^{2} \end{aligned}$$

4 $$\begin{aligned}&R^{2} = \frac{\text {TSS}-\text {ESS}}{\text {TSS}},\,(0\le R^{2}\le 1) \end{aligned}$$

5 $$\begin{aligned}&\text {VIF}=\frac{1}{1-R^{2}} \end{aligned}$$

where *N* is the number of participants, $Y_{i}$ is a value of variable *i*, $\hat{Y_{i}}$ is the predicted value of the ordinary least square (OLS) regression model that is trained to predict $Y_{i}$ using the other variables, and $\bar{Y_{i}}$ is the mean of the variable *i*. TSS measures the variation of $Y_{i}$ and ESS measures the difference between $Y_{i}$ and $\hat{Y_{i}}$ (i.e., error of the OLS). When $Y_{i}$ has a linear relationship with other variables, ESS decreases and becomes 0, $R^{2}$ increases to 1, and VIF becomes large. Thus, we can conclude that a large value of VIF indicates $Y_{i}$ linearly related with other variables^27^. We considered that a variable has multicollinearity if its VIF is greater than 4^28^. We removed multicollinearity by sequentially excluding variables with the largest VIF from the dataset until all the variables had a VIF smaller than 4.

In addition, we applied the Boruta algorithm to further remove irrelevant variables^29^. First, we concatenated the original variables and its randomly shuffled variables (shadow variables) into one dataset. Second, we trained the RF to classify CVD and measured the variable importance using SHAP (SHAP is described in a following subsection). Third, we repeated this process 1000 times and counted the number of hitting times that each original variable had a greater SHAP than a maximum importance shadow variable (MISV), $t_{\text {hitting}}$. Finally, we performed a binomial test to verify whether the each original variable is statistically significant than MISV to CVD classification. For the binomial test, we established the null hypothesis $H_{0}$: the expected hitting probability of an original variable is 50% (i.e., the variable and MISV have the statistically same importance), as well as the alternative hypothesis: $H_{a}$: the expected hitting probability of an original variable is greater than 50% (i.e., the variable is statistically more important than MISV). We tested these hypotheses with right-side significance test:

6 $$\begin{aligned}&p_{\text {right}}=\text {Binomial}(t>t_{\text {hitting}},\,T=1000,p=0.5) \end{aligned}$$

7 $$\begin{aligned}&\text {Significance level:}\,p_{\text {right}}<\frac{0.05}{M} \end{aligned}$$

where *p* is the success probability in the binomial distribution, *t* is the number of successes, *T* is the total number of trials, and *M* is the number of variables after VIF filtering. Bonferroni correction was applied to the significance level with $\alpha$ = 0.05^30^. If the $p_{\text {right}}$ is lower than $\frac{0.05}{N}$, we accepted the alternative hypothesis and used the variable as the input for the CVD classifier. The Boruta selection implies that the selected variables are significantly more related to CVD than the randomly generated variables (shadow variables)^29^.

### Training of machine learning models

After the variable selection, we trained four ML models for CVD prediction: MLP, SVM, RF, and L-GBM. We applied 5-fold cross-validation and grid search for hyperparameter tuning. Cross-validation (CV) is an efficient technique to prevent overfitting by validating the model with the various training and validation data. First, we randomly split the train set into five folds and trained models on four out of five folds (training data). Next, we measured the area under the receiver operating characteristic (AUC) on the other fold (validation data). Then, we iterated this procedure five times while alternating validation data. We used the averaged AUC over five iterations as a CV score. We found optimal hyperparameters that have maximum CV score using grid-search:

8 $$\begin{aligned} \{(h_1,h_2,...,h_n)\in H|\forall (x_1,x_2,...,x_n)\in H : f(h_1,h_2,...,h_n)\ge f(x_1,x_2,...,x_n)\} \end{aligned}$$

where *H* is the cartesian product of the hyperparameters and *f* is the CV score.

The class proportion of the study population was extremely imbalanced (CVD:non-CVD $=$ 1:31.23). The model should overfit the non-CVD cases and achieve low sensitivity performance with imbalanced dataset. Therefore, we artificially balanced the ratio between CVD and non-CVD cases, making this ratio equal to 16.12:16.12 by using undersampling and synthetic minority oversampling technique (SMOTE) so that the model sufficiently train CVD patient cases^31^. These balancing algorithms were only applied in the training process but not in the validation or test. We used Scikit-Learn (https://scikit-learn.org) and Imbalanced-Learn (https://imbalanced-learn.org) to implement the model training.

### Variable importance

We conducted SHAP analysis to understand the influence of each variable on the prediction results and what are the main variables that affect the CVD prediction. SHAP is a model-agnostic feature importance method that does not temper the properties of the trained model. SHAP separately measures the contributions of each variable to the CVD prediction based on the Shapley value. SHAP also shows whether the risk factors positively or negatively impacted the prediction^20^. The Shapley value is defined as:

9 $$\begin{aligned} v_{i}=\sum _{S}\frac{\left| S \right| !(n-\left| S \right| -1)!}{n!}(p(C\mid S\cup \{x_{i}\})-p(C\mid S)) \end{aligned}$$

where $v_{i}$ is the Shapley value of variable $x_{i}$, *S* is the set of variables excluding $x_{i}$, *n* is the total number of variables, and $p(C\mid X)$ is the classifier output or the posterior probability that a participant has CVD given the set of variables X. The Shapley value quantifies the expected change on the posterior probability when the variable $x_{i}$ is excluded from the dataset. SHAP value approximates the Shapley value as the weight of a linear regression model. A positive SHAP value indicates the variable contributed to classifying a participant as part of the CVD class. Conversely, a negative value means the variable contributed to classifying a participant as belonging to the non-CVD class. We used the SHAP algorithm developed by Lundberg and Lee publicly available at https://github.com/slundberg/shap^20^.

### Performance metrics

The proposed models predict the posterior probability of a participant having CVD, given the selected variables. Classification means that the predicted sample is discriminated as positive or negative based on a threshold value. If the prediction is greater than the threshold, it is classified as CVD, and if it is less than the threshold, it is classified as non-CVD. Area under the receiver operating characteristic curve (AUC) is a widely used metric to indicate binary classification performance in medical data analysis^32^. The receiver operating characteristic (ROC) curve represents the trade-off between the false positive rate ($FPR=\frac{\text {True negative}}{\text {False positive}+\text {True negative}}=$ 1 - specificity) and the true positive rate ($TPR=\frac{\text {True positive}}{\text {True positive}+\text {False negative}}=$ sensitivity) by varying the prediction threshold from 0 to 1. Therefore, AUC is a threshold-independent performance metric. However, when using the classifier in practice, it is necessary to set a specific threshold. We found the optimal threshold ($\widehat{Th}$) that maximizes the geometrical mean of sensitivity and specificity (G-mean). The G-mean is a widely used metric to represent the balanced performance of sensitivity and specificity^33^. The threshold of each model $\widehat{Th}$ was optimized by:

10 $$\begin{aligned} \widehat{Th}=\underset{Th}{\text {argmax}}\sqrt{\text {sensitivity}(Th)\times \text {specificity}(Th)}. \end{aligned}$$

**Figure 2:**
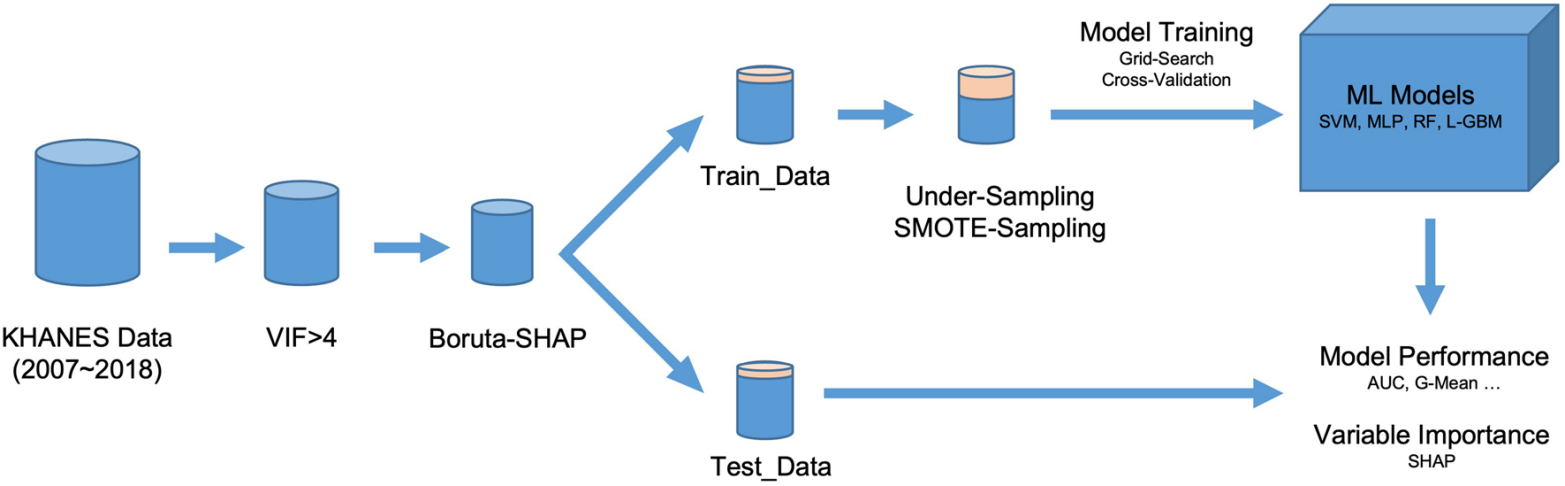
Graphical illustration of the study.

We represented the overall performance with the mean and 95% confidence intervals (CI) on the bootstrapping samples^34^. A graphical illustration of the study methods is shown in Fig. 2.

### Ethics statement

Approval of the research: The institutional review board (IRB) of Kyung Hee University Medical Center approved the study (IRB No. KHUH 2020-06-054). All methods were carried out in accordance with the KNHANES analytic guidelines and regulations. Informed consent: All subjects provided written informed consent before participating in this survey (KNHANES).

**Table 1:** General characteristics of selected variables.

Variables	Non-CVD (n $$=$$ 210,846)	CVD (n $$=$$ 6752)	P-value
Age, years, mean (SD)	46.61 (16.32)	66.26 (9.97)	<0.001
Monthly income, $, mean (SD)	3191.78 (2480.18)	2093.86 (2334.61)	<0.001
ALT, IU/L, mean (SD)	22.29 (19.6)	23.15 (14.52)	<0.001
AST, IU/L, mean (SD)	22.55 (13.33)	25.1 (16.04)	<0.001
BMI, kg/m2, mean (SD)	23.71 (3.49)	24.7 (3.17)	<0.001
HBsAg, IU/L, mean (SD)	139.06 (879.32)	98.97 (745.63)	<0.001
Height, m, mean (SD)	163.66 (9.47)	159.98 (9.42)	<0.001
Red blood cell count, mil/uL, mean (SD)	4.63 (0.48)	4.48 (0.49)	<0.001
Urine glucose, mean (SD)	0.09 (0.51)	0.26 (0.86)	<0.001
White blood cell count, thous/uL, mean (SD)	6.23 (1.75)	6.44 (1.9)	<0.001
**Frequency of drinking**			<0.001
Not at all in the past year, n (%)	51,591 (24.47%)	3132 (46.39%)	
Less than 1 time a month, n (%)	37,962 (18.0%)	864 (12.8%)	
1 time a month, n (%)	21,690 (10.29%)	492 (7.29%)	
2-4 times a month, n (%)	49,876 (23.66%)	910 (13.48%)	
2-3 times a week, n (%)	33,599 (15.94%)	729 (10.8%)	
More than 4 times a week, n (%)	14,997 (7.11%)	544 (8.06%)	
**Drinking amount**			<0.001
No drinking, n (%)	51,591 (24.47%)	3132 (46.39%)	
1-2 shots, n (%)	52,223 (24.77%)	1577 (23.36%)	
3-4 shots, n (%)	33,874 (16.07%)	905 (13.4%)	
5-6 shots, n (%)	25,573 (12.13%)	382 (5.66%)	
7-9 shots, n (%)	23,110 (10.96%)	389 (5.76%)	
More than 10 shots, n (%)	23,565 (11.18%)	286 (4.24%)	
**Weight change in the past year**			<0.001
Weight loss, n (%)	29,120 (13.81%)	1344 (19.91%)	
Weight maintenance, n (%)	132,275 (62.74%)	4492 (66.53%)	
Weight gain, n (%)	48,389 (22.95%)	815 (12.07%)	
**Stress perception level**			<0.001
Little, n (%)	31,573 (14.97%)	1669 (24.72%)	
A little, n (%)	120,381 (57.09%)	3229 (47.82%)	
Much, n (%)	47,836 (22.69%)	1345 (19.92%)	
Very much, n (%)	9987 (4.74%)	419 (6.21%)	
**Urine protein**			<0.001
−, n (%)	176,293 (83.61%)	5365 (79.46%)	
±, n (%)	16,255 (7.71%)	573 (8.49%)	
+, n (%)	2088 (0.99%)	153 (2.27%)	
++, n (%)	740 (0.35%)	85 (1.26%)	
+++, n (%)	254 (0.12%)	25 (0.37%)	
++++, n (%)	27 (0.01%)	5 (0.07%)	
Anemia, n (%)	15,942 (7.56%)	891 (13.2%)	<0.001
Diabetes mellitus, n (%)	17,648 (8.37%)	1963 (29.07%)	<0.001
High cholesterol, n (%)	28,365 (13.45%)	2019 (29.9%)	<0.001
Hypertension, n (%)	55,894 (26.51%)	4458 (66.02%)	<0.001
Irregular pulse, n (%)	2894 (1.37%)	412 (6.1%)	<0.001
Marriage status, n (%)	1671,13 (79.26%)	6606 (97.84%)	<0.001
Sex (female), n (%)	111,709 (52.98%)	3030 (44.88%)	<0.001
Successful high school graduate status, n (%)	150,397 (71.33%)	2028 (30.04%)	<0.001

Continuous variables were expressed as a mean value (standard deviation), while discrete (except binary) and binary variables were expressed as the number of participants, n (percentage, %). We calculated the P-values of continuous, discrete, and binary variables using t-test, Mann-Whitney, and chi-square techniques, respectively. A two-tailed P-value (<0.001) was considered statistically significant. In representing monthly income, 1172 KRW was converted to 1 USD.

## Results

### Variable selection and general characteristics

This study initially extracted 34 variables that are related to CVD from KNHANES^24^. We provide a complete list of initial and selected variables in Supplementary Table S1. The weight, waist circumference, hematocrit, and hemoglobin were filtered out according to the condition of VIF< 4. In addition, the smoking amount, exercise frequency, hepaB, hypertriglyceridemia, urine ketone, urine bilirubin, and urine nitrite were identified as CVD-irreverent variables by the Boruta algorithm. As a result, 23 variables were selected to develop the CVD classifiers. We defined the study population by applying complex sampling weights to reduce bias in the original dataset. The general characteristics of the study population with selected variables are presented in Table 1. Among a total of 217,598 participants, 6752 (3.1%) participants had CVD. Compared with participants in the non-CVD group, participants with CVD had higher age, ALT, AST, BMI, white blood cell, and urine protein. Also, the participants with CVD had a higher rate of anemia, diabetes mellitus, high cholesterol, hypertension, and irregular pulse than the non-CVD group. On the contrary, monthly income, HBsAg, red blood cell count, drinking frequency, drinking amount, successful high school graduate status, and sex (female) were higher in the non-CVD group.

### Comparison of model performance

**Figure 3:**
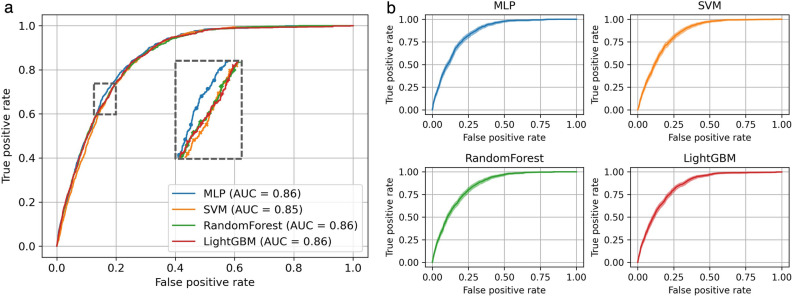
(**a**) ROC curves of CVD classifiers. (**b**) ROC curves with the confidence interval. The bootstrap mean is represented by lines, and shadows represent the confidence interval.

Table 2 describes the performances of the different approaches for CVD classification. The *S* and *B* indicate variable selection and class balancing, respectively. At first, we compared the performances of each CVD classifier by applying variable selection, class balancing and $\widehat{Th}$. The MLP model achieved the highest AUC (0.862 [95% CI, 0.854–0.869]) followed by the RF model (0.857 [95% CI, 0.849–0.865]). The SVM model achieved the lowest AUC (0.853 [95% CI, 0.845–0.860]). The ROC curves also show that the MLP model has the largest AUC (Fig. 3). We also compared the sensitivity, specificity, and G-mean. The SVM model achieved the highest sensitivity (0.849 [95% CI, 0.812–0.902]), and was the best model for classifying the positive class (CVD patients). However, the specificity of the SVM model (0.727 [95% CI, 0.681–0.762]) was the lowest among the tested models. In contrast, the MLP model achieved the highest specificity (0.745 [95% CI, 0.691–0.773]), which means it was best for classifying the negative class (non-CVD participants). The G-mean showed that the MLP model was the best classifier when considering negative and positive classes together (0.792 [95% CI, 0.783–0.802]). Next, we analyzed the impact of variable selection, and class balancing approaches (i.e., SMOTE and random undersampling) through the performance of MLP. Feature selection did not cause a significant change in overall performance while class balancing decreased $\widehat{Th}$ significantly. This result indicates that the classifier trained on imbalanced data, thereby overfitting the non-CVD class. As shown in Table 2, we observed that with a threshold of 0.5, the G-mean and sensitivity decreased significantly, and the specificity increased to 1 when class balancing was removed. In summary, feature selection had little effect on the performance of the model, but imbalanced data made the model overfit the non-CVD class. Supplementary Tables S2 and S3 show the complete list of classification performance. All the performances were calculated with the optimal hyperparameters found by five-fold CV and grid-search. We provide the optimal hyperparameters and their grids in Supplementary Table S4.

**Table 2:** Classification performance with comparing the effect of variable selection and class balancing techniques.

Method	$\widehat{Th}$	AUC	G-mean	Sensitivity	Specificity
**Performance comparison of different models**					
SVM+*S*+*B*	0.351	0.853 (0.845–0.860)	0.786 (0.776–0.795)	**0.849 (0.812–0.902)**	0.727 (0.681–0.762)
RF+*S*+*B*	0.385	0.857 (0.849–0.865)	0.787 (0.777–0.797)	0.846 (0.806–0.889)	0.732 (0.693–0.766)
L-GBM+*S*+*B*	0.360	0.856 (0.848–0.864)	0.790 (0.780–0.799)	0.839 (0.814–0.866)	0.744 (0.725–0.756)
MLP+*S*+*B*	0.455	**0.862 (0.854–0.869)**	**0.792 (0.783–0.802)**	0.842 (0.810–0.897)	**0.745 (0.691–0.773)**
***S*** **and/or *B*** **ablation performance**					
MLP+*B*	0.43	0.857 (0.849–0.864)	0.787 (0.778–0.797)	0.837 (0.805–0.874)	0.740 (0.708–0.77)
MLP+*S*	0.026	0.849 (0.841-0.857)	0.780 (0.771–0.789)	0.853 (0.817–0.893)	0.714 (0.675–0.744)
MLP	0.033	0.857 (0.849–0.864)	0.788 (0.778–0.798)	0.839 (0.808–0.877)	0.740 (0.699–0.76)
***S*** **and/or** ***B*** **ablation performance with default threshold**					
MLP+*S*+*B*	0.5	0.862 (0.854–0.869)	0.790 (0.779–0.8)	0.816 (0.795–0.837)	0.764 (0.760–0.768)
MLP+*B*	0.5	0.857 (0.849–0.864)	0.782 (0.770–0.793)	0.791 (0.769–0.813)	0.773 (0.769–0.777)
MLP+*S*	0.5	0.849 (0.841–0.857)	0.112 (0.083–0.138)	0.013 (0.007–0.019)	0.998 (0.998–0.998)
MLP	0.5	0.857 (0.849–0.864)	0.045 (0–0.072)	0.002 (0–0.005)	0.999 (0.999–1)

All performance metrics are described as bootstrap mean and 95% confidence interval. The bold values indicate the best classification performance when variable selection and class balancing both applied: variable selection *S*, class balancing *B*.

### Risk factor analysis

**Table 3:** Ranking of variable importance for CVD classification.

Variables	MLP +*S*+*B*	SVM+*S*+*B*	RF+*S*+*B*	L-GBM+*S*+*B*	*Avg*+*S*+*B*	*Avg*+*S*	*Avg*+*B*	*Avg*
Age	0.213	0.192	0.14	0.194	0.185	0.028	0.156	0.027
Sex (female)	0.078	0.06	0.039	0.036	0.053	0.013	0.043	0.014
Hypertension	0.053	0.053	0.063	0.043	0.053	0.008	0.051	0.007
Successful high school graduate status	0.014	0.022	0.036	0.008	0.020	0.007	0.025	0.003
Drinking amount	0.015	0.033	0.015	0.015	0.020	0.005	0.024	0.003
BMI	0.022	0.018	0.009	0.015	0.016	0.005	0.003	0.004
Monthly income	0.010	0.023	0.018	0.010	0.016	0.004	0.019	0.004
High cholesterol	0.024	0.014	0.012	0.008	0.015	0.005	0.014	0.004
Drinking frequency	0.012	0.018	0.013	0.015	0.014	0.005	0.015	0.005
Stress perception level	0.019	0.017	0.004	0.016	0.014	0.005	0.009	0.003
Red blood cell count	0.022	0.016	0.006	0.009	0.013	0.004	0.014	0.004
Diabetes mellitus	0.012	0.009	0.016	0.010	0.012	0.003	0.011	0.004
ALT	0.001	0.010	0.012	0.017	0.010	0.001	0.008	0.002
Height	0.003	0.010	0.008	0.011	0.008	0.004	0.007	0.004
AST	0.005	0.006	0.006	0.005	0.006	0.002	0.007	0.002
Weight change in the past year	0.005	0.012	0.003	0.001	0.005	0.002	0.011	0.002
Marriage status	0.010	0.001	0.008	0	0.005	0.002	0.003	0.001
White blood cell count	0.002	0.007	0.004	0.005	0.005	0.001	0.006	0.002
HBsAg	0.002	0.003	0.003	0.005	0.003	0.002	0.003	0.001
Irregular pulse	0.006	0.001	0.002	0.002	0.003	0.003	0.002	0.001
Urine protein	0.002	0.006	0.001	0	0.002	0.001	0.003	0.001
Urine glucose	0.002	0.005	0	0.001	0.002	0.001	0.001	0.001
Anemia	0	0.001	0.002	0.004	0.002	0.001	0.002	0.001

The variables are in descending order according to the mean value of the four classification models: variable selection *S*, class balancing *B*, average over the models *Avg*.

SHAP measures the contribution of each variable to the posterior probability from an individual prediction. We averaged the absolute value of SHAP over the test set to identify the top risk factor of CVD. We sorted the importance of variables in descending order according to the average value over the four models (i.e., *Avg*+*S*+*B* column of Table 3). The result shows that age was the most influential risk factor for CVD classification (0.185). The second and third were sex (0.053) and hypertension (0.053). Next, successful high school graduate status (0.020), drinking amount (0.020), BMI (0.016), and high cholesterol (0.015) followed sequentially. On the contrary, anemia (0.002), urine glucose (0.002), and urine protein (0.002) had minor effects on the CVD classification. The impact of class balancing and variable selection on SHAP analysis was significant. SHAP became very small when class balancing was ablated. This is because SHAP quantifies the importance of variables depending on the output of models (The predicted probability that the sample is a CVD patient). The imbalanced data lowered the output, thereby reducing SHAP values. In addition, the results showed that if we did not perform feature selection, multicollinearity occurred in the data, and the major risk factors (age, sex and hypertension) were underestimated. The results are shown in Table 3.

SHAP can be positive or negative depending on whether the variable contributed to classifying the participant as belonging to the CVD or non-CVD class. Table 4 shows the directionality of the top 10 contribute variables. The age, hypertension, BMI, high cholesterol, and stress perception level were positively correlated with CVD. This means that the higher the values of these variables, the more likely the model will classify the participant as a CVD patient. On the contrary, sex (female), successful high school graduate status, drinking amount, monthly income, and drinking frequency were negatively correlated with SHAP, and participants with lower values of these variables are likely to be classified as a CVD patient. We also calculated Pearson coefficients between the input variables and the outcome variable, the CVD (Supplementary Table S5). The directionality analysis of SHAP and the Pearson coefficients were consistent except for the stress perception level. Stress perception had a very weak negative correlation (−0.02) with CVD according to the Pearson coefficient, but classifiers used the stress perception as a CVD-positive variable.

**Table 4:** Correlation coefficients between variables and SHAP.

Variables	MLP	SVM	RF	L-GBM
Age	+0.98	+0.98	+0.95	+0.95
Sex (female)	−0.95	−0.96	−0.97	−0.92
Hypertension	+0.96	+0.96	+0.98	+0.89
Successful high school graduate status	−0.95	−0.94	−0.97	−0.86
Drinking amount	−0.96	−0.95	−0.94	−0.70
BMI	+0.97	+0.87	+0.86	+0.84
Monthly income	−0.94	−0.94	−0.84	−0.54
High cholesterol	+0.96	+0.94	+0.98	+0.81
Drinking frequency	−0.96	−0.86	−0.93	−0.72
Stress perception level	+0.91	+0.84	+0.56	+0.73

The values were calculated using a model with variable selection, class balancing, and optimal hyperparameters applied.

## Discussion

The prevalence of CVD is continuously increasing due to the influence of westernized dietary habits and health behaviors, and accompanying complications and mortality are also increasing^35,36^. As policy interest in chronic diseases with high socioeconomic cost increases, it has become a major task of public health to discover and manage people with risk factors before the onset of the disease as an effective prevention method^37^. Therefore, based on KNHANES, this study confirmed the prevalence of CVD in adults over 20 years of age in Korea, and identified the risk factors for CVD associated with demographic factors, comorbid factors, lifestyle factors, and physiological factors. We also suggested correlations between risk factors and their contributions on CVD classification.

This study developed several risk assessment models to characterize the risk of CVD using ML methods. All the models achieved high predictive performance with AUCs ranging from 0.853 to 0.862. Compared to other models, the MLP model showed the best performance with an AUC value of 0.862 (AUC, 0.862 [95% CI 0.854–0.869]), and the model’s performance was significantly better than the existing risk score^38^. Our research has shown that ML technology is a successful standout when it comes to identifying important risk factors in large-scale epidemiological studies. Previous studies have demonstrated the important role of ML in other medical fields, such as T2DM, obesity, and heart failure^9,39,40^. Our results confirmed the outstanding performance of ML in CVD risk assessment. This is the first study that evaluates the importance of variables using various ML methods with KNHANES data and checks the risk factors of CVD. Previous study in recent years has shown that machine learning improves prediction accuracy in cardiovascular event prediction and these methods may lead to greater insights regarding subclinical disease markers^41^. The most recent development has shown that the ML Risk Calculator outperformed the ACC/AHA Risk Calculator by recommending less drug therapy and additional studies are underway to validate the ML model in other cohorts and to explore its ability in short term CVD risk prediction^42^. In addition, recently, the ML model for predicting CVD events in asymptomatic individuals was built using data for more than 400,000 UK participants^10^. The most predictive non-laboratory variables were ages, gender, smoking status, usual walking pace, self-reported overall health rating, previous diagnoses of high blood pressure, income, and parents’ ages at death^10^. Most CVD risk factors are consistent with previous results. However, our result shows that smoking status was not an important variable, and gender was the second CVD risk factor. Our results were more fairly derived because the previous study used an empirical permutation method (Alaa, Bolton et al. 2019). In contrast, we used SHAP, which has a solid theoretical foundation in game theory. There is a similar previous study in that they also analyzed Korean national data and used SHAP for CVD risk factor analysis^16^. However, we provided a more intuitive interpretation. We designed the ML to output posterior probabilities of CVD, *p*(CVD|*X*) while other studies have different scales of SHAP for each model. We can intuitively interpret our results (Table 3) as the average change in the CVD prediction probability that is caused by each variable. Also, We further investigated the directionality between risk factors and CVD using SHAP. Age, hypertension, and BMI positively affect CVD risk, whereas sex (female), alcohol consumption, and income had a negative effect on CVD risk.

Our risk factor analysis suggests that VIF filtering was a useful technique for removing multicollinearity and increasing the interpretability of the models. We observed some CVD risk factors were clarified by applying the variable selection. For example, BMI has been reported as a significant risk factor for CVD^43,44^. However, as shown in *Avg*+*B* column of Table 3, the importance of BMI was underestimated to 0.003. This is because weight and waist circumference have collinearity with BMI, leading to offsetting the SHAP of the BMI. We removed those variables as VIF< 4, thereby correcting the importance of BMI to 0.016. Also, the VIF filtering affects correcting underestimated risk factors (e.g., age, sex, and Hypertension) and overestimated risk factors (e.g., successful high school graduate status and drinking amount). We concluded that removing multicollinearity based on VIF is helpful to reduce the error of SHAP-based risk factor analysis.

Feature selection is a commonly used technique to reduce computational costs and prevent models from overfitting. Some studies analyzed the effect of filter-based feature selection, such as chi-square, ReliefF, and SU on heart disease classification^45–47^. Those studies have demonstrated that variable selection improved or degraded the classification performance depending on the model. Compared to these studies, we used a model-based selection algorithm, i.e., the Boruta, which conducts statistical significance tests for variable selection. As shown in Table 2 and Supplementary Table S2, our feature selection also improved or degraded prediction performances depending on which model was used. However, we demonstrated that Boruta correctly distinguished irrelevant variables from CVD-relevant variables. The P-values in Table 1 show that all the selected variables had significantly different distribution between CVD patients and normal people.

We also observed the imbalanced data decreased sensitivity when we used default thresholds (Table 2). This is because imbalanced data led the model to overfit the majority class (non-CVD) and significantly reduced the prediction of ML. We solved overfitting by applying class balancing techniques. However, if the goal is only to achieve higher sensitivity, adjusting the classification threshold is an easier way than applying a class balancing technique. Because the sampling rate of SMOTE or undersampling is also a hyperparameter and tuning them is another burden for data scientists. In contrast, the threshold adjustment does not require additional hyperparameter tuning and has a lower computational cost than the balancing techniques. The results show that we achieved high sensitivity only by adjusting the threshold (MLP+*S* row in Table 2). However, Table 3 shows that class balancing was essential to clarify the importance of risk factors. The small SHAP value made it challenging to compare which variable is more important. Class balancing magnified SHAP values and made it easier to distinguish variable importance.

We also analyzed the effect of the interaction of variables on CVD prediction with SHAP. Supplementary Fig. S1 shows the interaction between age and alcohol consumption on CVD. According to the plot, for participants under 40 years of age, the SHAP values go sideways then increase linearly from 40. Our analysis shows that if the age factor is over 40, the participants are more likely to be classified as CVD patients. However, since age is a factor beyond the clinician’s control, it can be emphasized that there is a need to pay attention to adults who can manage modifiable and important risk factors, such as their alcohol consumption, BMI, and cholesterol. As shown in Supplementary Fig. S2, the effect of drinking frequency is dependent on age (the red dots on the plot represent the SHAP of participants with high alcohol consumption). For participants under the age of 60, there was no relationship between alcohol consumption and the SHAP of age. However, for participants over 60 years of age, those who drink more tend to have higher SHAP values. Controlling the drinking frequency of the population over 60 years of age can lower the prevalence of CVD. In conclusion, our analysis can provide the probability of CVD and individual risk factors for each group or patient, which can be used in preventive management of CVD.

Limitations of this study should be addressed. First, since it is a cross-sectional study that analyzed data without a follow-up investigation of participants, there is a limit in explaining the direction of the causal relationship between risk factors and CVD. Second, to evaluate the generalization performance, future research should be conducted with external verification. Third, when defining CVD, the presence of disease was judged through the subject’s self-response. In order to increase the validity of the responses, we defined the presence of disease using the phrasing ‘Diagnosed by a doctor and currently suffering from the disease,’ but there are still some limitations in validity. Fourth, we could not exclude the influence of people who have already started CVD treatment. In our future work, this effect can be removed by excluding these CVD patients.

In conclusion, this study investigated the prevalence of cardiovascular disease, which has a very high disease burden, based on the latest data from KNHANES (i.e., a representative sample survey for the entire Korean population). Also, this study applied ML and the model interpretation technique to identify risk factors that can be reversibly controlled to prevent CVD. Our results show that ML can correlate many variables with individual diseases based on a wide range of data. This provides integrated insights into the multivariate risk factors of CVD.

## Supplementary Information

Supplementary Information.

## Data Availability

The datasets analysed during the current study are available in the KNHANES repository, https://knhanes.kdca.go.kr/knhanes/main.do.
