# Supplementary material for: Machine learning-based diagnosis and risk factor analysis of cardiocerebrovascular disease based on KNHANES
Source: Sci Rep. 2022 Feb 10;12:2250. doi: 10.1038/s41598-022-06333-1 (PMC8831514; doi:10.1038/s41598-022-06333-1)
Supplement: Supplementary file 1 — Supplementary Information. [file 41598_2022_6333_MOESM1_ESM.docx]

**Machine Learning-based Diagnosis and Risk Factor Analysis of Cardiocerebrovascular Disease Based on KNHANES**

**Taeseob Oh, Dongkyun Kim, Siryeol Lee, Changwon Won, Sunyoung Kim, Ji-soo Yang, Junghwa Yu, Byungsung Kim, and Joohyun Lee**

**Supplementary Table 1.** Results of variable selection

| **Data type** | **Variable** | **VIF filtering** | **Boruta selection** |
| --- | --- | --- | --- |
| **Continuous** | Age |  |  |
|  | Monthly income |  |  |
|  | Weight | X |  |
|  | Height |  |  |
|  | Waist circumference | X |  |
|  | BMI |  |  |
|  | HBsAg |  |  |
|  | AST |  |  |
|  | ALT |  |  |
|  | Hemoglobin | X |  |
|  | Hematocrit | X |  |
|  | White blood cell |  |  |
|  | Red blood cell count |  |  |
|  | Urine glucose |  |  |
| **Ordinal** | Smoking amount |  | X |
|  | Drinking frequency |  |  |
|  | Drinking amount |  |  |
|  | Exercise frequency |  | X |
|  | Stress perception level |  |  |
|  | Weight change in the past year |  |  |
|  | Urine ketone |  | X |
|  | Urine bilirubin |  | X |
|  | Urine protein |  |  |
| **Binary** | Sex |  |  |
|  | Marriage status |  |  |
|  | Successful high school graduate status |  |  |
|  | Irregular pulse |  |  |
|  | Urine nitrite |  | X |
|  | Diabetes mellitus |  |  |
|  | High cholesterol |  |  |
|  | Hypertriglyceridemia |  | X |
|  | HepaB |  |  |
|  | Anemia |  |  |
|  | Hypertension |  |  |

This table describes which variables were selected to build machine learning models. An “X” means the variable is filtered out.

**Supplementary Table 2.** Comparison of cardiocerebrovascular disease classifiers performance with threshold optimization

| **Method** | **Threshold** | **AUC** | **G-mean** | **Sensitivity** | **Specificity** |
| --- | --- | --- | --- | --- | --- |
| **MLP** | 0.0326 | 0.857 [0.849 - 0.864] | 0.788 [0.778 - 0.798] | 0.839 [0.808 - 0.877] | 0.740 [0.699 - 0.76] |
| **MLP+*S*** | 0.0262 | 0.849 [0.841 - 0.857] | 0.780 [0.771 - 0.789] | 0.853 [0.817 - 0.893] | 0.714 [0.675 - 0.744] |
| **MLP+*B*** | 0.4298 | 0.857 [0.849 - 0.864] | 0.787 [0.778 - 0.797] | 0.837 [0.805 - 0.874] | 0.740 [0.708 - 0.77] |
| **MLP+*S*+*B*** | 0.4553 | 0.862 [0.854 - 0.869] | 0.792 [0.783 - 0.802] | 0.842 [0.810 - 0.897] | 0.745 [0.691 - 0.773] |
| **SVM** | 0.0424 | 0.740 [0.728 - 0.752] | 0.677 [0.666 - 0.688] | 0.727 [0.653 - 0.758] | 0.631 [0.613 - 0.7] |
| **SVM+*S*** | 0.0459 | 0.736 [0.723 - 0.748] | 0.678 [0.664 - 0.69] | 0.679 [0.638 - 0.731] | 0.678 [0.625 - 0.715] |
| **SVM+*B*** | 0.3612 | 0.851 [0.844 - 0.859] | 0.787 [0.777 - 0.796] | 0.842 [0.819 - 0.88] | 0.736 [0.695 - 0.743] |
| **SVM+*S*+*B*** | 0.3509 | 0.853 [0.845 - 0.86] | 0.786 [0.776 - 0.795] | 0.849 [0.812 - 0.902] | 0.727 [0.681 - 0.762] |
| **RF** | 0.0466 | 0.859 [0.851 - 0.867] | 0.793 [0.783 - 0.802] | 0.850 [0.819 - 0.874] | 0.739 [0.722 - 0.763] |
| **RF+*S*** | 0.0478 | 0.861 [0.853 - 0.868] | 0.792 [0.783 - 0.801] | 0.850 [0.814 - 0.885] | 0.738 [0.708 - 0.766] |
| **RF+*B*** | 0.3495 | 0.858 [0.851 - 0.866] | 0.787 [0.779 - 0.796] | 0.870 [0.824 - 0.904] | 0.712 [0.681 - 0.748] |
| **RF+*S*+*B*** | 0.3854 | 0.857 [0.849 - 0.865] | 0.787 [0.777 - 0.797] | 0.846 [0.806 - 0.889] | 0.732 [0.693 - 0.766] |
| **L-GBM** | 0.0642 | 0.854 [0.846 - 0.861] | 0.785 [0.777 - 0.795] | 0.834 [0.800 - 0.88] | 0.740 [0.700 - 0.762] |
| **L-GBM+*S*** | 0.055 | 0.852 [0.845 - 0.86] | 0.784 [0.775 - 0.794] | 0.841 [0.791 - 0.898] | 0.732 [0.677 - 0.774] |
| **L-GBM+*B*** | 0.3767 | 0.857 [0.849 - 0.865] | 0.788 [0.779 - 0.798] | 0.829 [0.797 - 0.872] | 0.751 [0.717 - 0.774] |
| **L-GBM+*S*+*B*** | 0.3602 | 0.856 [0.848 - 0.864] | 0.790 [0.780 - 0.799] | 0.839 [0.814 - 0.866] | 0.744 [0.725 - 0.756] |

This is the classification performance when the threshold is optimized to maximize the G-mean. All performance metrics are described as bootstrapping averages and 95% confidence intervals: variable selection *S*, class balancing *B*.

**Supplementary Table 3.** Comparison of cardiocerebrovascular disease classifiers performance without threshold optimization

| **Method** | **Threshold** | **AUC** | **G-mean** | **Sensitivity** | **Specificity** |
| --- | --- | --- | --- | --- | --- |
| **MLP** | 0.5 | 0.857 [0.849 - 0.864] | 0.045 [0.000 - 0.0717] | 0.002 [0.000 - 0.0051] | 0.999 [0.999 - 1.0] |
| **MLP+*S*** | 0.5 | 0.849 [0.841 - 0.857] | 0.112 [0.083 - 0.138] | 0.013 [0.007 - 0.0191] | 0.998 [0.998 - 0.998] |
| **MLP+*B*** | 0.5 | 0.857 [0.849 - 0.864] | 0.782 [0.770 - 0.793] | 0.791 [0.769 - 0.813] | 0.773 [0.769 - 0.777] |
| **MLP+*S*+*B*** | 0.5 | 0.862 [0.854 - 0.869] | 0.790 [0.779 - 0.8] | 0.816 [0.795 - 0.837] | 0.764 [0.760 - 0.768] |
| **SVM** | 0.5 | 0.740 [0.728 - 0.752] | 0.044 [0.000 - 0.0709] | 0.002 [0.000 - 0.0050] | 1.000 [1.000 - 1.0] |
| **SVM+*S*** | 0.5 | 0.736 [0.723 - 0.748] | 0.000 [0.000 - 0.0] | 0.000 [0.000 - 0.0] | 1.000 [1.000 - 1.0] |
| **SVM+*B*** | 0.5 | 0.851 [0.844 - 0.859] | 0.753 [0.740 - 0.766] | 0.698 [0.673 - 0.723] | 0.813 [0.809 - 0.816] |
| **SVM+*S*+*B*** | 0.5 | 0.853 [0.845 - 0.86] | 0.767 [0.754 - 0.78] | 0.731 [0.707 - 0.756] | 0.805 [0.801 - 0.809] |
| **RF** | 0.5 | 0.859 [0.851 - 0.867] | 0.000 [0.000 - 0.0] | 0.000 [0.000 - 0.0] | 1.000 [1.000 - 1.0] |
| **RF+*S*** | 0.5 | 0.861 [0.853 - 0.868] | 0.000 [0.000 - 0.0] | 0.000 [0.000 - 0.0] | 1.000 [1.000 - 1.0] |
| **RF+*B*** | 0.5 | 0.858 [0.851 - 0.866] | 0.761 [0.747 - 0.774] | 0.708 [0.683 - 0.733] | 0.817 [0.813 - 0.821] |
| **RF+*S*+*B*** | 0.5 | 0.857 [0.849 - 0.865] | 0.760 [0.747 - 0.774] | 0.710 [0.686 - 0.736] | 0.814 [0.810 - 0.818] |
| **L-GBM** | 0.5 | 0.854 [0.846 - 0.861] | 0.000 [0.000 - 0.0] | 0.000 [0.000 - 0.0] | 1.000 [1.000 - 1.0] |
| **L-GBM+*S*** | 0.5 | 0.852 [0.845 - 0.86] | 0.000 [0.000 - 0.0] | 0.000 [0.000 - 0.0] | 1.000 [1.000 - 1.0] |
| **L-GBM+*B*** | 0.5 | 0.857 [0.849 - 0.865] | 0.745 [0.731 - 0.759] | 0.666 [0.642 - 0.691] | 0.832 [0.829 - 0.836] |
| **L-GBM+*S*+*B*** | 0.5 | 0.856 [0.848 - 0.864] | 0.747 [0.733 - 0.76] | 0.676 [0.651 - 0.701] | 0.825 [0.821 - 0.829] |

This is the classification performance with default threshold of 0.5. All performance metrics are described as bootstrapping averages and 95% confidence intervals: variable selection *S*, class balancing *B*.

**Supplementary Table 4.** Grid set and optimal hyperparameters

| **Classifier** | **Hyperparameter grid** | **Optimal** |
| --- | --- | --- |
| **MLP** | {solver: [‘adam’, ‘sgd’],  hidden_layer_sizes: [(20, 20), (40, 40), (60, 60), (20, 20, 20), (40, 40, 40), (60, 60, 60), (80, 80, 80), (100, 100, 100)],  activation: [‘logistic’, tanh’, ‘relu’],  alpha: [0.0001, 0.0003, 0.0006, 0.0009]} | {solver: ‘adam’,  hidden_layer_sizes: (40, 40, 40),  activation: ‘logistic’  alpha: 0.0006} |
| **SVM** | {C: [10^(numpy.linspace(-2,2,20))],  kernel: [‘rbf’, ‘sigmoid’],  gamma: [‘scale’, ‘auto’, 0.1, 1, 10]} | {C: 0.298,  kernel: ‘rbf’,  gamma: ‘auto’} |
| **RF** | {n_estimator: [100, 200, 400, 700, 1000],  max_depth: [4 ,6, 8, 10, 12],  min_samples_split: [5, 10, 30, 50, 70, 100],  min_samples_leaf: [5, 10, 30, 50, 70, 100]} | {n_estimator: 400,  max_depth: 12,  min_samples_split: 30,  min_samples_leaf: 10} |
| **LGBM** | {boosting_type: [‘gbdt’, ‘dart’],  max_depth: [8 ,9],  min_child_samples: [10, 20, 40, 60, 80, 100],  num_leaves: [120, 170, 250, 300, 350, 400],  learning_rate: [0.1, 0.05],  subsample: [0.6, 0.8]} | {boosting_type: ‘dart’,  Max_depth: 9,  Min_child_samples: 100,  Num_leaves: 120,  Learning_rate: 0.05,  Subsample: 0.6] |

The name of each hyperparameter is the same as the attribute name of the Scikit-Learn library (https://scikit-learn.org/).

**Supplementary Table 5.** Correlation coefficient between input variables and CVD

| **Variables** | **Correlation coefficient** |
| --- | --- |
| Age | 0.21 |
| Sex (female) | -0.03 |
| Hypertension | 0.15 |
| Successful high school graduate status | -0.16 |
| Drinking amount | -0.08 |
| BMI | 0.05 |
| Monthly income | -0.08 |
| High cholesterol | 0.09 |
| Drinking frequency | -0.06 |
| Stress perception level | -0.02 |

This is the correlation coefficient between the selected input variables and CVD. The correlation coefficient of the numeric variable was calculated using the point bi-serial, and the binary variable was calculated using the Phi coefficient.

**Supplementary Figure 1.** Dependency plot between age and drinking frequency.


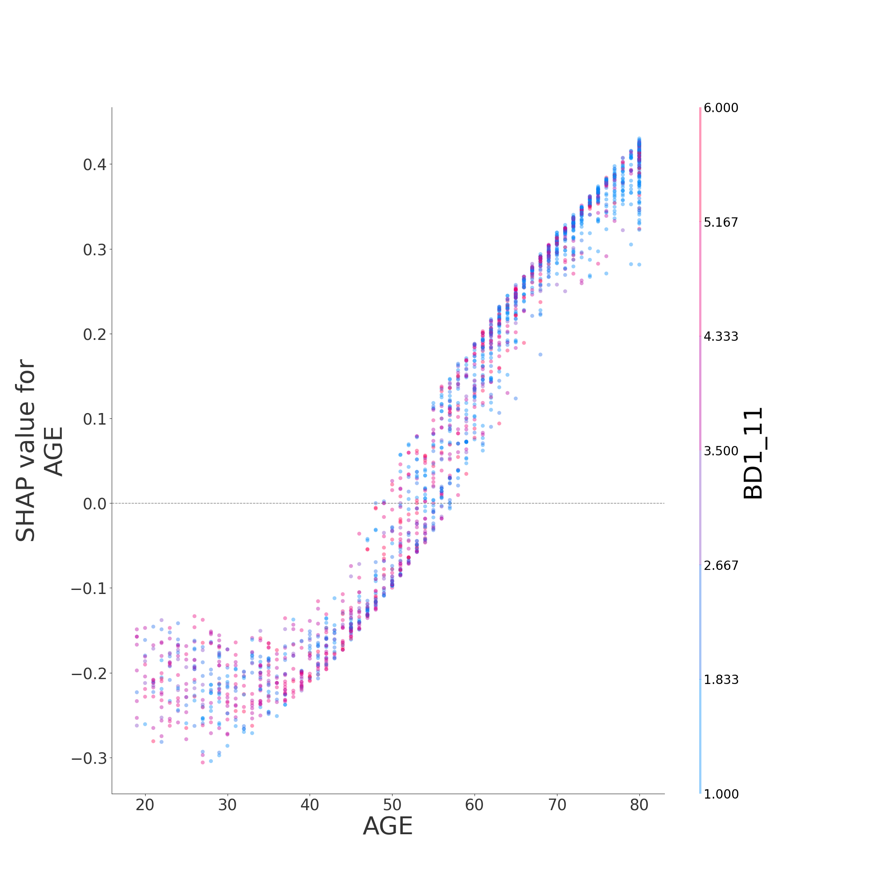


This figure shows the dependence between age and CVD in SHAP. The figure also shows the interaction between age and drinking frequency. Red dots indicate high drinking frequency, and blue dots indicate low drinking frequency. BD1_11 is the original code name of the drinking frequency in KNHANES.
